# The brine depth of the Khorat Basin in Thailand as indicated by high-resolution Br profile

**DOI:** 10.1038/s41598-021-88037-6

**Published:** 2021-04-21

**Authors:** Lijian Shen, Nuchit Siritongkham, Licheng Wang, Chenglin Liu, Anont Nontaso, Wanitchaya Khadsri, Yufei Hu

**Affiliations:** 1 MNR Key Laboratory of Metallogeny and Mineral Assessment, Institute of Mineral Resources, Chinese Academy of Geological Sciences, 26 Baiwanzhuang Road, Xicheng District, Beijing, 100037 China; 2 Mineral Resources Division, Department of Mineral Resources, Ministry of Natural Resources and Environment, Bangkok, 10400 Thailand; 3 CAS Center for Excellence in Tibetan Plateau Earth Sciences, Key Laboratory of Continental Collision and Plateau Uplift, Institute of Tibetan Plateau Research, Chinese Academy of Sciences, Beijing, 100101 China

**Keywords:** Marine chemistry, Geochemistry

## Abstract

Bromine contents of a 17-cm halite core from drilling hole of K203 in the Khorat Basin were analysed at 1 cm intervals (17 samples in total). The Br contents range from 99 to 184 ppm with a rapid variation. The K/Mg ratios of halite samples are tens of times higher than those of primary halite fluid inclusions. There is no positive correlation between Mg and Br contents, suggesting that fluid inclusions impose very little or negligible influence on Br contents of halites. The Br contents are not controlled by potash minerals either because the SEM examination shows no potash minerals and there is no relationship between K and Br contents. The Br contents of halite are thus primarily controlled by the Br concentrations of parent brines. The rapid variation of Br contents of halite within this section suggests a shallow saline pan wherein the giant Khorat evaporites were formed. This is contradictory to previous Br profiles of the Lower Salt Member which showed relatively stable and continuously increasing trends. The shallow saline pan model evidenced by high-resolution Br profile is consistent with sedimentary facies and salt mineral textures.

## Introduction

The Cretaceous to early Tertiary evaporites are present in both the Khorat and the Sakon Nakhon sub-basins, in Maha Sarakham Formation on the Khorat Plateau of southeast Asia. The evaporites of the Maha Sarakham Formation lie atop of a thick non-marine sequence of the Mesozoic Khorat Group and are intercalated with non-marine red beds^1^. The Maha Sarakham Formation contains more than 1000 m of salt layers (anhydrite, halite and potash salts)^1,2^. Ancient evaporite deposits are usually much thicker than modern ones^3^, for instance, > 1100 m of evaporite salts of Zechstein sequence, Germany; > 600 m of anhydrite and halite of Permian deposits of Texas, USA^3^. A deep-basin model was proposed to interpret the formation of exceptionally thick ancient evaporite sequences^3^. However, there are no active depositional areas where saline giants are being formed on the world’s surface^4^, therefore the deep-basin model could not be validated.

The principle for identifying the depth of brine pool is as follows: rapid change in Br content of halite layer on cm-scale indicates shallow salt-pan environment and vice versa^5^. If the standing body of brine was shallow, the reduction/elevation of brine level would cause rapid changes in Br contents of precipitated halites. On the contrary, the Br content of precipitated halite wouldn’t show significant variations when the brine was rather deep because the net evaporation or inflow caused negligible effect on the composition of brine.

The Br profiles from several halite sections (hundreds of metres thick) in the Maha Sarakham Formation showed a relatively stable and slowly increasing trend from the bottom to the top^1^. The resolution of those profiles is low because the sampling intervals were several meters^1^. If the variation of cm-scale Br content is consistent with the those of meter-scale Br content^1^, a deep-water model (at least not too shallow) would be applicable. However, in this case, it would be in contradiction to the evidence of sedimentary facies and textures of salt minerals which indicated that a shallow saline pan was present during the deposition of evaporite^1^; whereas a shallow-water model would be corroborated when the variation of cm-scale Br contents shows a rapid change, which is not consistent with those of meter-scale Br contents.

In this study, we measured the Br contents of cm-scale halite intervals in the Khorat Basin in order to reveal whether or not a deep basin pool existed during the deposition of evaporite.

## Geologic setting

**Figure 1:**
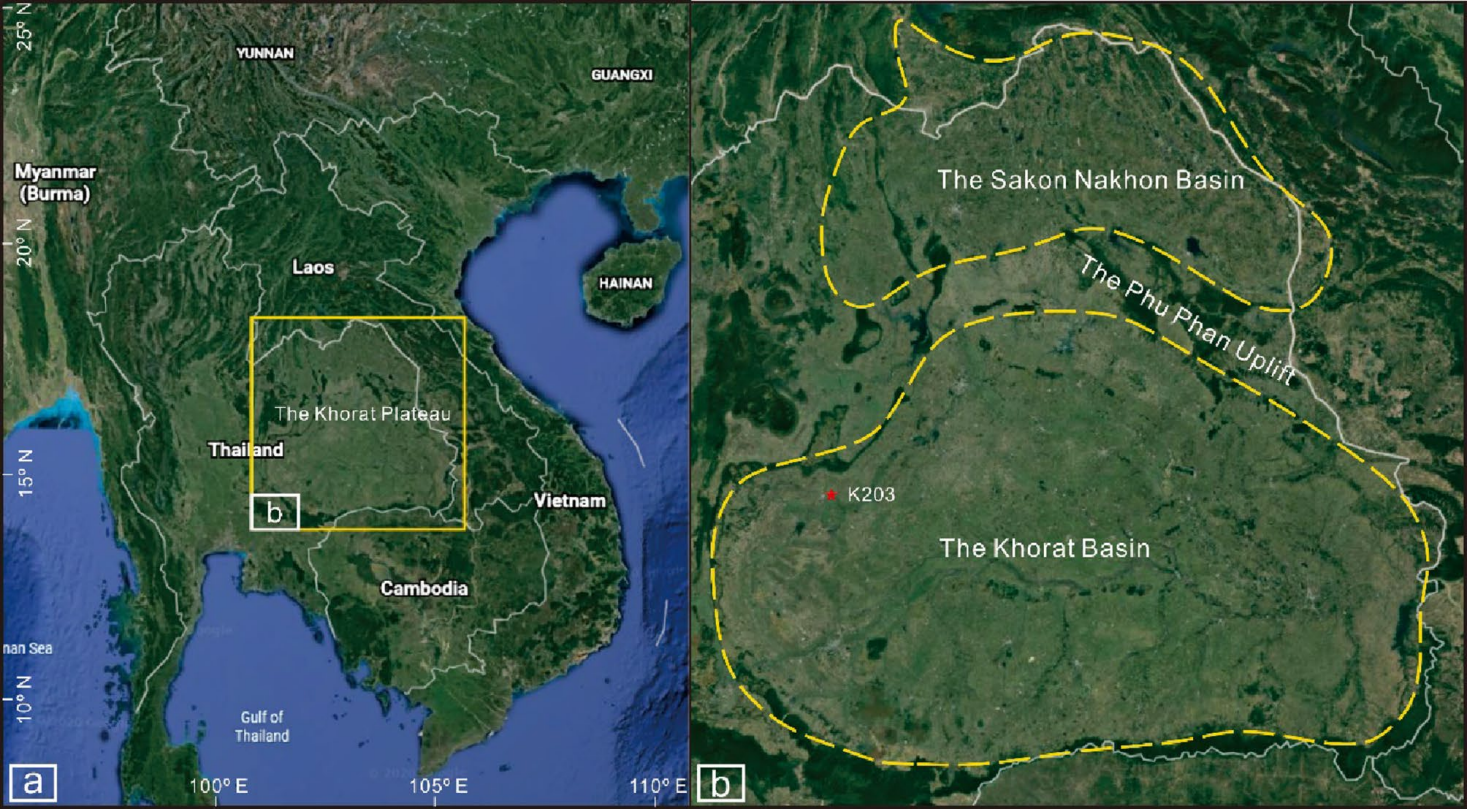
The distribution of the Khorat Basin and Sakon Nakhon Basin on the Khorat Plateau (modified from Google Earth). (**a**) The location of the Khorat Plateau; (**b**) the distribution of the Sakon Nakhon Basin and the Khorat Basin^7^.

The Khorat Basin and Sakon Nakhon Basin are located on the Khorat Plateau (Fig. 1a), separated by the Phu Phan Uplift (Fig. 1b). The Khorat Plateau is a broad synclinorium and lies between latitudes 14° and 19° N and between longitudes 101° and 106° E^6^.

The evaporites within the Khorat Plateau are in the Maha Sarakham Formation^2^. The Maha Sarakham Formation is composed of evaporites and continental red-bed deposits, and mostly overlain by a thin layer of Tertiary alluvium^1,8^. The thickness of this formation is about 250 m on average, with a maximum of over 1 km in the centre of the basin. Typically, from bottom to top, the Maha Sarakham Formation could be divided into several members, namely the Basal Anhydrite Member, the Lower Salt Member, the Potash Layer, the Lower Clastic Member, the Middle Salt Member, the Middle Clastic Member, the Upper Salt Member and, the Upper Clastic Member^1^.

The Basal Anhydrite Member is found throughout the Khorat and Sakon Nakhon basins over a total area of 170,000 km^2^. The thickness of this member is relatively consistent, ranging from 0.9 to 1.5 m. The contact between the anhydrite unit and the overlying halite layer is sharp and unconformable. The Lower Salt Member contains anhydrite nodules throughout the formation. Thin anhydrite layers and siliciclastic mud layers are interbedded with the Lower Salt Member. The Potash Layers occurs in the top section of the Lower Salt Member. The Lower Clastic Member which overlies the potash layer is a reddish to brown mudstone. The mudstone layers are interbedded with rare siltstone layers. Clastic matrix with irregular masses of halite crystals is common. The Middle Salt Member directly overlies the Lower Clastic Member and is composed of well-bedded halite layers. The halite layers are interbedded with thin anhydrite layers. The Middle Clastic Member consists of massive red to purple claystone and silty mudstone. In most cases, the Upper Salt Member is absent in the Khorat Plateau due to its shallow burial depth and wet climate; It seems that at least half the salt of this member has been leached and dissolved. The Upper Clastic Member represents the uppermost sedimentary layer of the Maha Sarakham Formation. The thickness of this member is highly variable, up to 680 m. The Upper Clastic Member consists of pale reddish-brown silty claystones and sandstones and cross-beds are commonly developed. Some layers contain well-defined root traces^1^.

## Materials and methods

**Figure 2:**
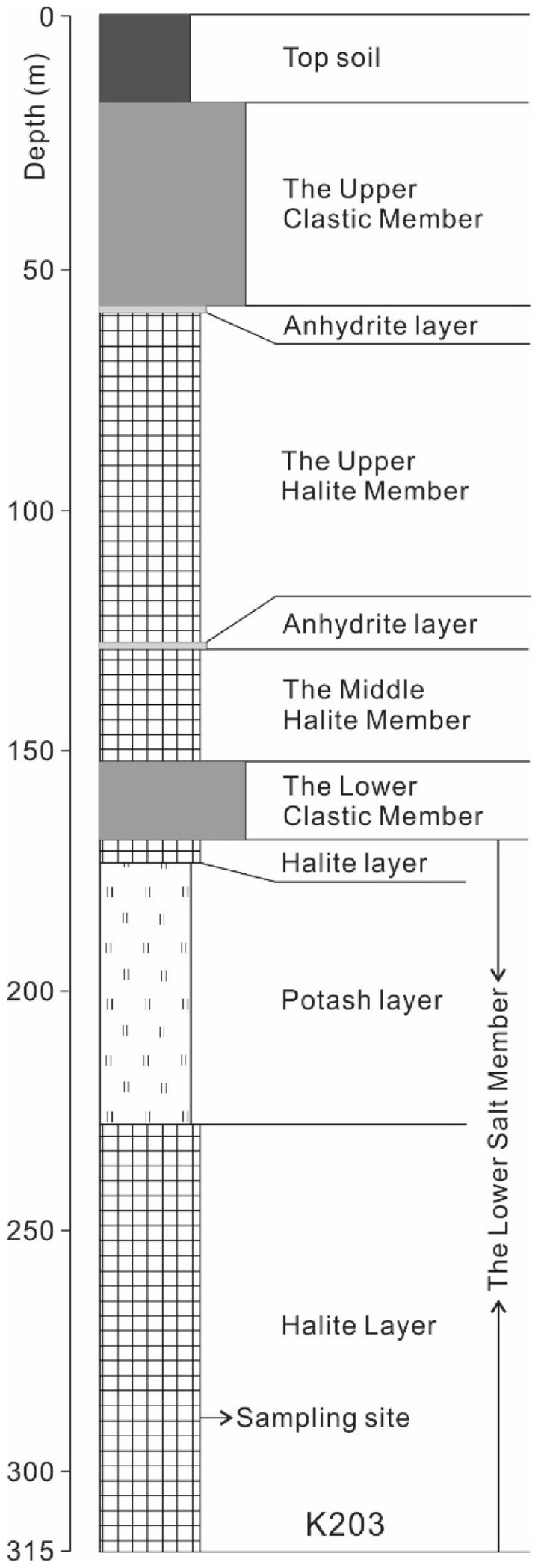
The schematic column of K203 and the sampling site ( modified from the Department of Mineral Resource, Thailand).

The halite sections (17 cm in length) was collected from Hole K203 (located at Chaiyaphum, Thailand, Fig. 1) at a depth of 285 m within the Lower Salt Member (Fig. 2). The evaporite sequence in the Hole K203 consists of three salt members: The Lower, Middle and Upper Salt Members.

The selected core section was cut in half. One half has been archived; the other half was split into 17 sampling intervals of 1 cm each. All samples were dissolved with distilled water.

Br, Cl, Na, K, Mg and Ca are analysed using Inductively Coupled Plasma Optical Emission Spectrometry, SPECTRO, German at the MNR Key Laboratory of Metallogeny and Mineral Assessment. The detailed analysis procedure was described in previous study^9^. The analytical uncertainty is ± 5% for Na and Cl, and ± 3% for K, Mg, Ca and Br, respectively.

The pure halite and anhydrite-bearing halite samples were examined by SEM. The SEM analysis was carried out at the Key Laboratory of Deep-Earth Dynamics, Institute of Geology, Chinese Academy of Geological Sciences, using the FEI Nova NanoSEM 450. The backscattered electron (BSE) images were taken under operating voltage of 15–20 kV and the working distance of 13.5 mm.

## Results

### Salt minerals

**Figure 3:**
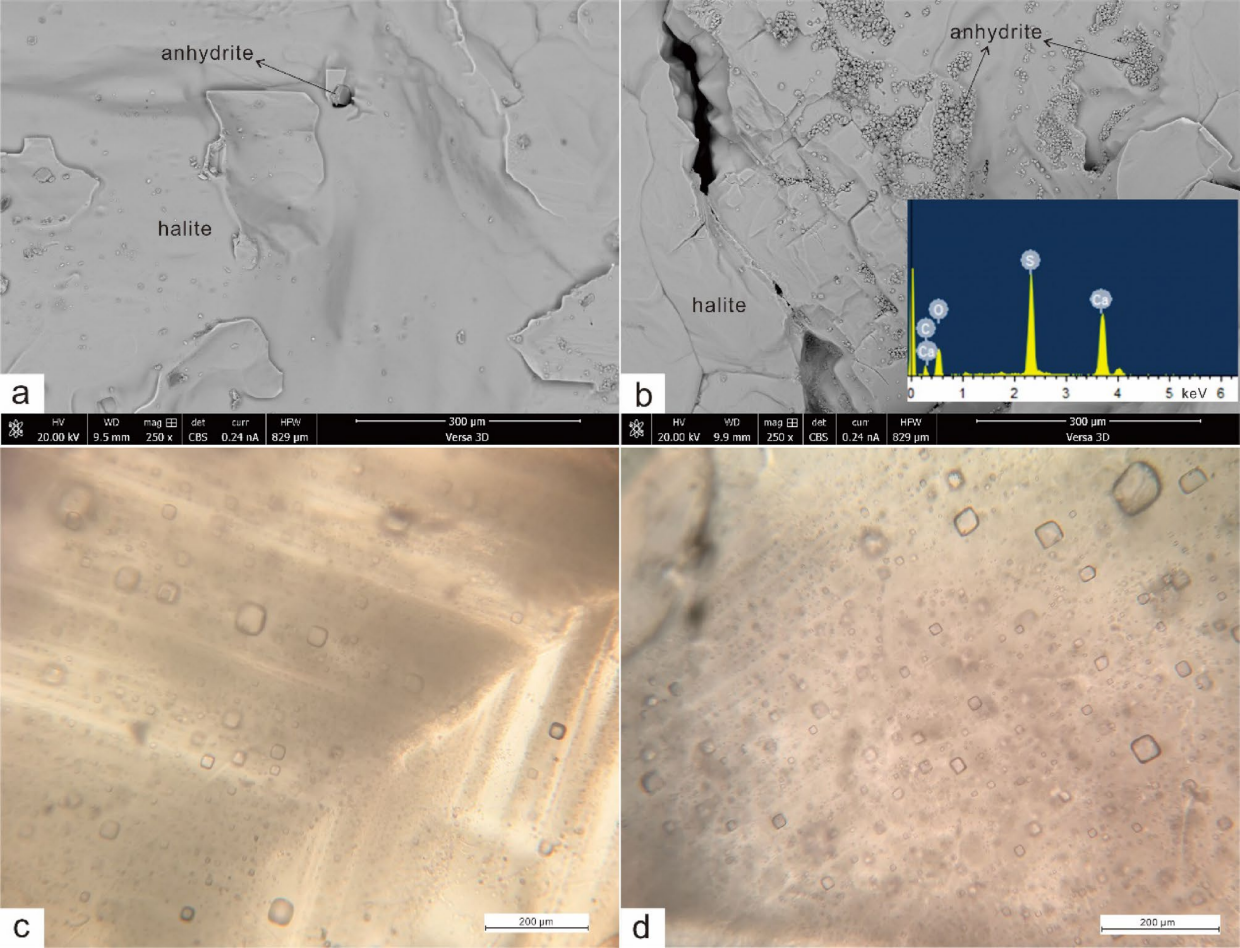
SEM images of evaporite samples and characteristics of fluid inclusions in halite (**a**) pure halite; (**b**) halite with trace amount of anhydrite (inset shows EDS analysis of fine-grained anhydrite); (**c**) chevron halite crystal; (**d**) hopper halite crystal.

SEM microphotographs show that the halite sections consist of nearly pure halite, with a trace amount of anhydrite (Fig. 3a). The anhydrite-bearing sections mainly contain halite crystals, and anhydrites are usually present as fine-grained aggregates (Fig. 3b). The halite samples contain cloudy, euhedral crystals with chevron (Fig. 3c) and hopper features (Fig. 3d) outlined by primary fluid inclusions.

### Major and trace elements

Na and Cl account for major components of all samples. The total of Na, Cl, K, Mg, Ca and Br ranges from 94.11 to 106.39%, which is consistent with the uncertainties (5% for Na, 3% for Cl, K, Mg, Ca, and Br). All samples contain a rather low amount of K, Mg, Ca and Sr. This is consistent with the SEM evidence that all samples are mainly composed of halite.

**Figure 4:**
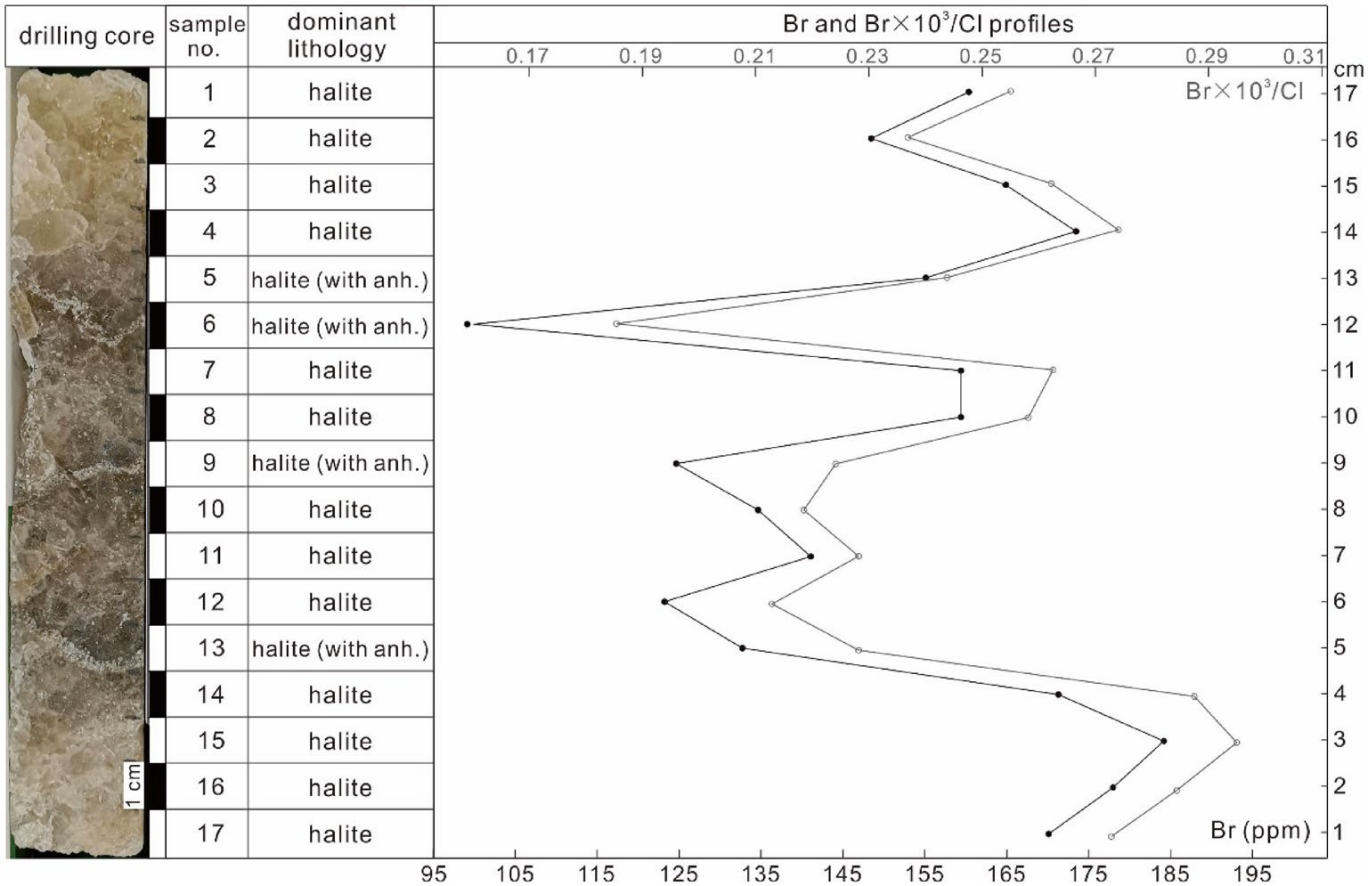
The bromine profile of a halite interval from a drilling core in the Khorat Basin. *anh.* anhydrite; solid circle: Br content; hollow circle: Br × 10^3^/Cl ratio.

The Br contents of halites range from 99 to 184 ppm. Three low Br content troughs are observed in this section, corresponding to three anhydrite-bearing halite layers. In pure halite intervals, the Br contents are higher than 145 ppm (Table 1, Fig. 4).

**Table 1:** K, Mg, Ca, Br, Na and Cl contents of halite samples from the Hole K203.

Sample ID	K	Mg	Ca	Br	Na	Cl	Total	K/Mg	Br × 10^3^/Cl
	%	%	%	ppm	%	%			
K203-1	0.036	0.014	0.328	160	41.53	62.91	104.83	2.63	0.254
K203-2	0.039	0.020	0.335	148	41.94	62.64	104.99	1.94	0.236
K203-3	0.038	0.016	0.156	164	42.29	62.88	105.40	2.31	0.261
K203-4	0.033	0.008	0.287	173	42.79	63.25	106.39	4.09	0.274
K203-5	0.031	0.002	0.092	155	42.23	63.58	105.95	13.44	0.244
K203-6	0.031	0.011	4.384	99	35.92	53.75	94.11	2.70	0.184
K203-7	0.031	0.007	1.183	159	40.30	60.79	102.33	4.48	0.262
K203-8	0.032	0.012	0.412	159	40.51	61.77	102.75	2.80	0.257
K203-9	0.032	0.012	3.494	124	36.78	55.59	95.92	2.62	0.223
K203-10	0.035	0.012	0.752	134	40.88	61.60	103.29	2.79	0.218
K203-11	0.032	0.007	0.668	141	40.95	61.82	103.49	4.81	0.228
K203-12	0.034	0.011	2.141	123	38.25	57.92	98.37	3.05	0.212
K203-13	0.036	0.013	2.171	132	38.96	58.10	99.29	2.72	0.227
K203-14	0.033	0.010	1.499	171	40.18	59.63	101.37	3.31	0.287
K203-15	0.030	0.006	0.078	184	41.92	62.51	104.56	5.38	0.294
K203-16	0.040	0.012	0.053	178	42.13	62.62	104.87	3.28	0.284
K203-17	0.035	0.012	0.107	170	42.65	62.37	105.19	2.95	0.273

## Discussion

Bromine occurs generally exclusively with Cl in chlorides^10^. Sulfates and Cl-free borates have so far proved to be free of bromine^10^. However, a trace amount of Br might be present in liquid inclusions^10^. Bromine substitutes for chlorine when chloride precipitates from the parent brine. The substitution amount depends on the bromine concentration in the parent brine. Because the bromide ions have some difficulty in fitting into the chloride positions in the crystal lattice of the halite, the percentage of bromine entering into the crystal during growth is lower than its percentage in the solution^1^, namely, the partition coefficient (weight %Br(mineral)/weight %Br(solution)) is always less than 1.

The absorption capacity for Br by chlorides follows an order of sylvite > carnallite > halite^10^. At room temperature (25 °C), the partition coefficient of sylvite (0.73) is approximately ten times higher than that of halite (0.073). The partition coefficient of carnallite is 0.52, which is slightly lower than that of sylvite, but much higher than that of halite^10^. Consequently, if salt samples contain a certain amount of potash minerals, the Br content would be related to K and/or Mg contents, for instance, potash salts in the Mengyejing potash, Yunnan, China^11^.

The Br content of the first halite precipitated from the evaporation of seawater range from 65 to 75 ppm. At the late stage of evaporation, halite will contain 320 to 400 ppm Br when potash minerals precipitate^12^. The highest Br content (184 ppm) in this halite section indicates that the evaporation of brine did not reach the precipitation stage of potash minerals. Meanwhile, the SEM examination proves that only halite and anhydrite crystals are present in this section.

**Figure 5:**
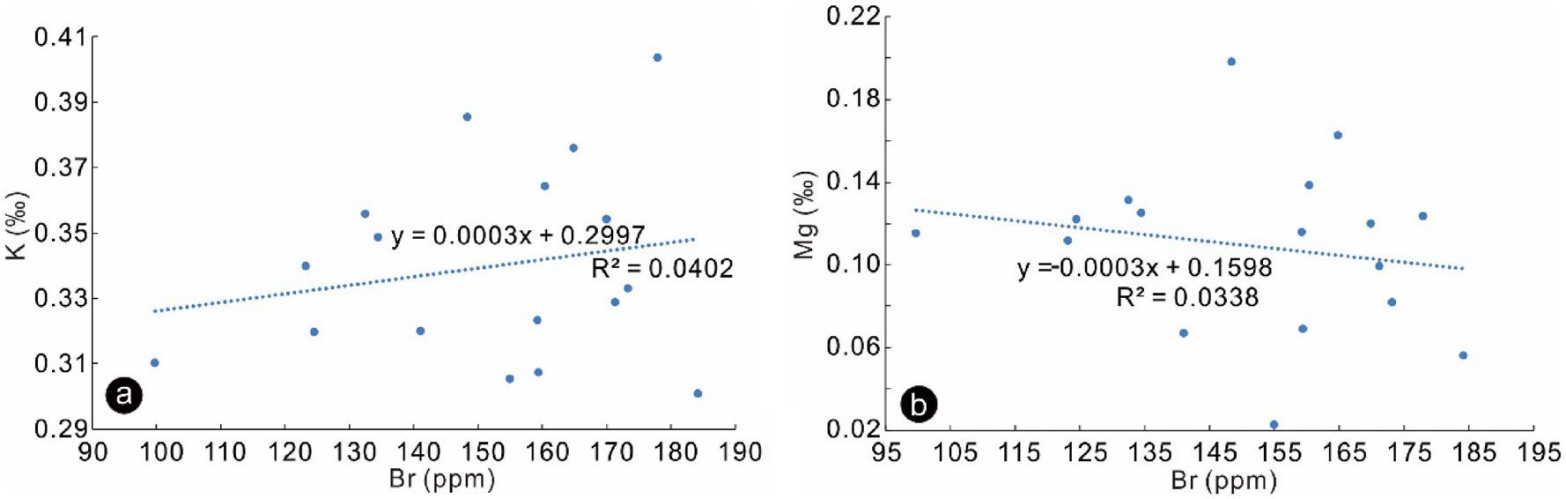
K and Mg vs. Br of halite samples from Hole K203, (**a**) K vs. Br; (**b**) Mg vs. Br.

The K and Mg contents of all samples are low and relatively stable (0.3 to 0.4‰ for K, 0.06 to 0.20‰ for Mg). Furthermore, there is no apparent relationship between K and Br contents (Fig. 5a). All evidence suggests that Br contents are not controlled by potash minerals.

K exists in halite in two forms: solid (crystal lattice, dislocations, and grain boundaries) and liquid (fluid inclusions). Br and K are present both in the solid and liquid phases of the halite, but Mg is exclusively in the fluid inclusions^13^. The primary fluid inclusions are visually abundant within halite crystals (Fig. 3c,d). If all K and Mg stem from fluid inclusions, the K/Mg ratios of halite samples should be consistent with those of halite fluid inclusions. The K/Mg ratios of halite samples in this study range from 1.19 to 8.27 (molar ratios). The K/Mg ratios of primary halite fluid inclusions in the Sakon Nakhon Basin (north part of the Khorat Plateau), Laos, show a range of 0.03–0.52^14^. Therefore, the K/Mg ratios of halite samples are much higher than those of halite fluid inclusions, suggesting that halite fluid inclusions have a negligible influence on the contents of trace elements in halite samples. Furthermore, if Br contents were dominated by halite fluid inclusions, the Br contents should be strongly related to the contents of Mg which exists exclusively in fluid inclusions^13^. The result shows no apparent correlation between Br and Mg (Fig. 5b), which indicates that the fluid inclusions exert no or negligible effect on Br contents of whole halite samples.

**Figure 6:**
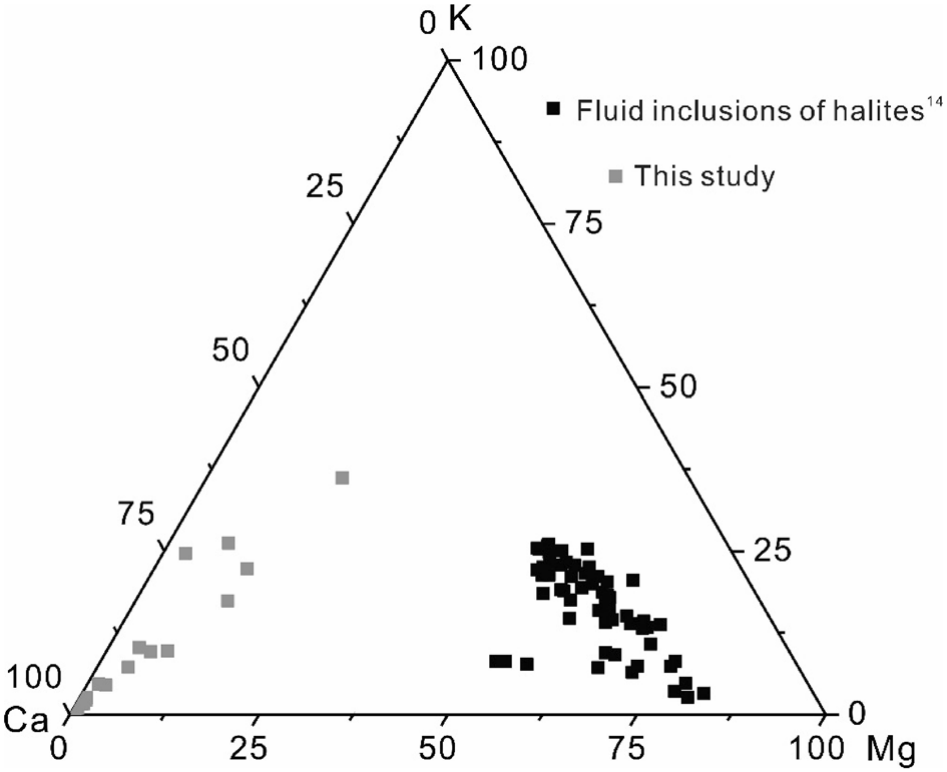
Compositions of halite fluid inclusions from the Maha Sarakham Formation^14^ and halite samples (this study) in the Mg–K–Ca ternary diagram.

The compositions of halite samples in this study depict a distinct Mg–K-Ca ternary diagram from that of halite fluid inclusions from the Maha Sarakham Formation (Fig. 6), which also denotes that those halite fluid inclusions exert minor or negligible influence on the integrated compositions of halite samples. Therefore, we propose that those fluid inclusions contribute trace amount of ion components, and the amount of Br within fluid inclusion has negligible or no influence on the integral Br content of halite samples.

In addition, the Br contents could also be affected by dissolution and recrystallization processes. Although halite samples show primary halite features (Fig. 3c,d), diagenetic alternation involving dissolution and recrystallization of halite could not be ruled out. Especially the salt domes and anticlinal structures are well-developed within the Khorat Basin^2,15^. Bromide frequently migrates into percolating brines during recrystallization^16,17^. Dissolution of ancient marine evaporites could produce either higher or lower Br/Cl ratios than the original brines, depending on whether early halites or late potash salts are dissolved^18^. The brine produced by the dissolution of early halite has lower Br/Cl ratio than that of seawater. This recycled brine would generate halite with low Br contents compared to the original one. This 17-cm long halite section was 60 m below the potash layers (Fig. 2). It was unlikely that the study section was affected by the dissolution of potash salts. If parts of this 17-cm halite section were formed by recrystallization, the recycled brine should have only derived from dissolution of original halite layer. The “recrystallised” basal halite (anhydrite-bearing halite) should contain Br content less than 75 ppm (Br content of first halite precipitated from seawater^10^), which is not the case. Therefore, we suggest that the extent of the dissolution–recrystallization process was very limited.

In conclusion, The Br contents of halite samples are mainly controlled by the concentration of parent brine in which halite was precipitated, rather than the “contamination” of potash minerals and fluid inclusions, or diagenetic alternations.

As mentioned above, all samples contain no or negligible K-Mg salts, and K and Mg occur in two forms, namely solid substitution and fluid inclusions. Due to the fact that halite crystals contain varied amounts of fluid inclusions and the extent of K–Mg substitution is unknown, K and Mg contents of halites could not be used as indicators for brine concentration. Only Br contents of halites can reflect the brine concentration exclusively.

The principle for estimating the brine pool depth is as follows: the halite-saturated brine produces 1 cm of halite with 8 cm reduction of brine^5^. If the depth of brine was very deep, the removal of 8 cm brine would not significantly alter the composition of brine (including Br concentration). The Br content of halite produced by deep brine should produce a very smooth profile, i.e., relatively stable or slightly upward increasing trend with progressive evaporation of brine^5^. An incipient study on the Khorat evaporite showed that the Br contents of the Lower Salt Member increased slowly from basal value of approximately 40 ppm to 60 ppm through around 85 m thickness^2^, while the sampling intervals were meters or decimetres. Subsequent study presented that the Br contents of halites in the Lower Member from four drill holes show a slow but continuous increase from the bottom (70–90 ppm) to the top (450 ppm)^1^. The lengths of halite drill cores from those four holes range from approximately 300 m to 600 m, with the sampling intervals of several meters. Their results showed that the Br contents of the Lower Salt Member increased slowly from the basal halite upward and did not change drastically. If cm-scale high-resolution of Br profile is in agreement with the Br profiles from previous studies^1,2^, a deep saline pan model would be suggested when applying to the principle^5^. However, the Br contents of halite samples within this 17 cm section vary drastically, showing a zigzag profile (Table 1, Fig. 4). Moreover, the trend of Br × 10^3^/Cl ratios of halites mimics the shape of Br profile (Fig. 4), which further support that the concentration of brine changed rapidly, because the degree of evaporation is closely related to Br content and Br × 10^3^/Cl ratios of halite^19^. Such rapid variations could eliminate the possibility of a deep brine pool in which the Khorat evaporites precipitated. It was with more possibility that the water-body in the Khorat Basin was very shallow, and a lowering/raising of brine level by several centimetres would change the halite Br content significantly. This is consistent with the conclusion that the Khorat evaporites were formed in a shallow saline-pan environment, based on sedimentary facies and textures of both halites and anhydrites^1^. The primary fabrics in halite beds (chevron halite structure) and halite-replaced gypsum and equant halite suggest a shallow saline pan environment^1^. The Br profile in this study shows a similar pattern to that of halite formed in the Mediterranean, which proposed that a shallow salt pan was prevailed during the Mediterranean salinity crisis^5^.

Why meter-scale Br profiles increased slowly and continuously is probably due to its low-resolution. The Br contents of meter-scale halite samples may have been derived from mixture of a certain intervals of halite section which failed to reflect minor variations of Br contents. With ongoing evaporation, the Br content of brine would be increasing progressively as a whole. The massive halites within the Lower Salt Member, not surprisingly, show a relatively stable and slowly increasing trend of Br content from the basal halite to the K-Mg salts layer in an overall pattern. The resolutions of the preceding studies on Br profiles are too low to detect the subtle changes of halite Br content, and only showed an overall trend of the whole salt member. Moreover, Br contents of halites with sampling intervals of 1–2 m showed a more varied profile^19^ compared with those with sampling intervals of meter-decameter^1,2^. Thus, the higher the sampling resolution is, the more accurate the fluctuation in Br content is obtained.

**Figure 7:**
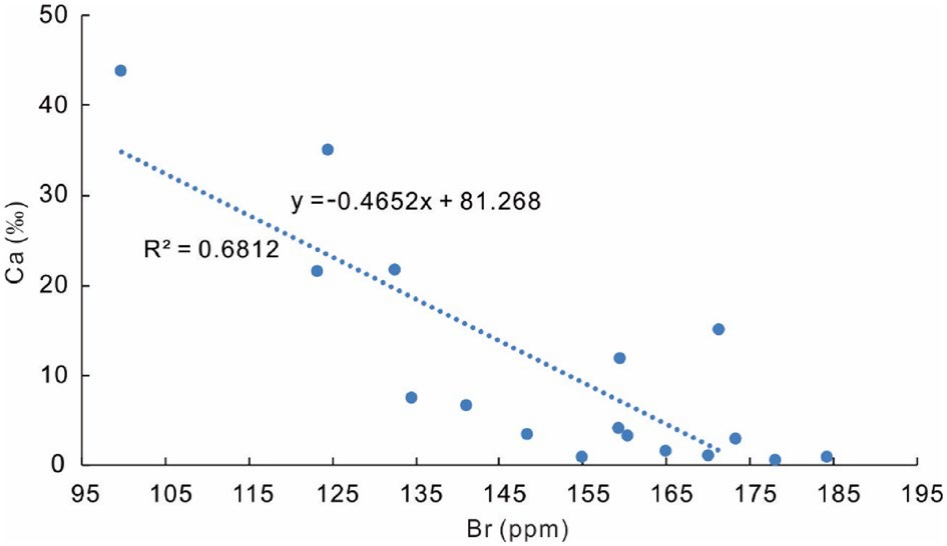
The relationship of Ca and Br contents of halite samples.

During the deposition of this 17 cm halite interval, the brine might have been diluted three times, corresponding to three anhydrite-bearing halite layers (Fig. 4). The Ca and Br contents display a reverse relationship (Fig. 7), indicating that more Ca-sulfates precipitation and lowering of Br concentration of brine occurred during dilution from freshwater inflow.

## Conclusion

Evidence from the Br contents of halite samples, the distance between the sampling site and the potash layer, the relationship between K and Br contents and, comparison between K/Mg ratios of halite and corresponding primary fluid inclusion indicate that the potash minerals and fluid inclusions have a rather limited or negligible influence on integral Br contents of halite from the Khorat Basin, and the occurrence of the dissolution–recrystallization process was unlikely. The Br contents of halites are controlled by the concentrations of parent brines. The rapid variation of Br contents within a 17-cm long halite interval suggests a shallow salt pan which is consistent with the sedimentary facies and salt minerals textures.
